# Effects of COVID-19 lockdowns on PhD candidates

**DOI:** 10.11604/pamj.supp.2020.35.2.25117

**Published:** 2020-07-27

**Authors:** Mbuzeleni Hlongwa

**Affiliations:** 1 Discipline of Public Health, School of Nursing and Public Health, University of KwaZulu-Natal, Durban, South Africa

**Keywords:** COVID-19, lockdowns, PhD

## To the Editors of the Pan African Medical Journal

Thousands of PhD candidates are facing challenges, personally and professionally, amid the COVID-19 pandemic, which has threatened populations across the globe. Many countries have, rightly, implemented lockdowns to reduce the rapid spread of COVID-19, as well as preparing healthcare systems to save lives. Tertiary institutions have been shut down. Working remotely from home has become a new reality for many, some of whom would come from rural areas, facing challenges such as poor network connectivity. This affects productivity. Progress for PhD candidates could be impacted negatively due to lockdowns because of COVID-19. Many of them rely on scholarship support to pursue research projects, pay for tuition fees, and are expected to produce high quality research outputs publishable to recognised and accredited journals. Generally, PhD programmes for full time students are expected to be completed within three academic years, four years in some cases. Therefore, scholarship support usually spans within the same period.

Lockdowns due to COVID-19 suggest that PhD candidates´ research projects activities such as stakeholder engagements or field work or laboratory experiments could be interrupted. Processing scholarship funding could be delayed or disrupted [1]. While some PhD candidates may be able to meet deadlines, depending on one´s level of study, many will be forced to consider extending study duration beyond the initially proposed period. This will likely result to additional fees for enrolment in universities. This will also delay them from progressing to early-career scientist positions such as post-docs. It is not clear at this point whether funding institutions will align with this shift to extend scholarship funding. To support PhD candidates, funding institutions should consider extending scholarship contracts to compensate for the extended period of study. PhD candidates often rely on conferences and other academic gatherings or congresses to meet and network with other scientists, present their work, find jobs, or collaborate. Many tertiary institutions have postponed awarding qualifications to thousands of deserving candidates, resulting to delayed PhD´s entry into the scientific space. While COVID-19 remain a threat globally, strategies and policies should be developed and implemented to support PhD´s entry to the research space and labour market.
